# Dextransucrase from the mutant of *Pediococcus pentosaceus* (PPm) is more stable than the wild type

**DOI:** 10.1007/s13205-011-0018-4

**Published:** 2011-07-30

**Authors:** Damini Kothari, Ankur Tyagi, Seema Patel, Arun Goyal

**Affiliations:** 1 Department of Biotechnology, Indian Institute of Technology Guwahati, Guwahati, 781 039 Assam India; 2 Department of Biotechnology, Lovely School of Sciences, Lovely Professional University, Jalandhar, 144402 India

**Keywords:** Pediococcus, Dextransucrase, Antibiogram, Carbohydrate, Fermentation, Stabilization

## Abstract

A comparative study on both wild type and mutant of *Pediococcus pentosaceus* for dextransucrase activity, its stability, dextran synthesizing activity, antibiotic sensitivity and carbohydrate utilization was performed. The wild type *P. pentosaceus* had specific activity of 0.58 U/mg whereas the mutant showed that of 1.0 U/mg with 72% enhancement. The antibiogram of 27 antibiotics tested against mutant showed significant differences with 9 antibiotics when compared to wild type. In carbohydrate fermentation profile, trehalose, galactose, maltose, lactose and fructose are metabolized by both the strains, but weakly in case of mutant. Stabilization of purified dextransucrase from wild type and mutant with various stabilizers was studied at 30 and 4 °C. Both enzymes were more stable at 4 °C. Among various stabilizers such as dextran (100 kDa, 10 μg/ml), glycerol (0.5%, *v/v*), PEG 8000 (10 μg/ml) and Tween 80 (0.5%, *v/v*), Tween 80 provided maximum stabilization at 4 and 30 °C. The mutant showed better stabilization than that of the wild type at both 30 and 4 °C. The loss of activity at 30 °C after 24 h in wild type and mutant in the presence of Tween 80 was only 34 and 32%, respectively, whereas the loss of activity in control of wild type and mutant was 76 and 59%, respectively. After 15 days at 4 °C, the loss of activity in control of wild type and mutant in the presence of Tween 80 was only 15 and 8%, respectively, whereas at 30 °C, the loss of activity in control of wild type and mutant was 49 and 42% respectively. Half-life of the enzyme with Tween 80 was 28.5 and 33.5 h for wild type and mutant, respectively, at 30 °C and 52.1 and 106.6 days for wild type and mutant respectively, at 4 °C.

## Introduction

Lactic acid bacteria (LAB) have a long history of safe use by man for food production and preservation. The LAB are widely used as starter cultures for fermentation in the dairy, meat and other food industries (Mugula et al. 2003). LAB can be used as cell factories for the production of an array of food additives and aroma compounds. Certain strains of *Lactococcus lactis* through their surface physicochemical properties interact and retain aroma compounds in food (Ly et al*.*^2008^). Fermentation of lupin protein extracts using several LAB improve their aroma (Schindler et al. 2011). The LAB may also function as probiotics and contribute to the general health of the consumer (Sybesma et al. 2006). Moreover, the LAB are known to synthesize enzymes, vitamins, antioxidants, bioactive peptides and bacteriocins (Fernandes et al. 1987; Knorr 1998). Several non-starter LAB produce bioactive peptides, generate gamma-aminobutyric acid and inactivate antigenotoxins, thus implicated in cheese-making (Settanni and Moschetti 2010). Many strains of *Lactobacillus* produce antifungal compounds acetic and phenyllactic acids to inhibit bread mold spoilage (Gerez et al. 2009). Antibacterial bacteriocin producing *Pediococcus pentosaceus* have been isolated from fermented sausages (Abrams et al. 2011). *Enterococcus faecium* has been reported as a potential producer of pediocin-like bacteriocin with antiviral activity (Todorov et al. 2010). The LAB have attracted immense commercial interests, for their capacity to secrete a wide range of exo-polysaccharides having industrially useful physico-chemical properties (Sidebotham 1974; De Vuyst and Degeest 1999; Ricciardi and Clementi 2000). The genera of LAB that produce dextrans using dextransucrase enzyme include *Streptococcus,Leuconostoc*, *Weissella* and *Lactobacillus* (Kralj et al. 2004; Tieking et al. 2005; Majumder and Goyal 2008). Smitinont et al*.* (1999) had emphasized on dextran synthesizing ability of the *Pediococcus* genus. Patel et al*.* (2010) reported the dextran production ability of *P. pentosaceus* for the first time. Dextrans are employed as blood plasma substitutes, plasminogen activators, antithrombogenic agents, in treatment of iron deficiency anaemia and in the matrix preparation of chromatography columns (Naessens et al*.*^2005^; Purama and Goyal 2005). Dextrans have major use in food formulations as stabilizing, emulsifying, texturizing and gelling agent. Dextran can be used as a stabilising coating to protect metal nanoparticles from oxidation and improve biocompatibility of biomaterials (Sengupta et al. 2006). The increase in the use of exo-polysaccharides in food, pharmaceutical and cosmetics industries emphasizes the importance of exploration of the new species and characterization of their traits. It has been reported that the genetic alterations of LAB that occur during random mutagenesis may lead to strains with improved traits (Sybesma et al. 2006). Mutants of *Leuconostoc* strains NRRL B-512F (Kim and Robyt 1994), B-742 (Kim and Robyt 1995a, b), B-1299 (Kim and Robyt 1995a, b) and 512FMC (Kitaoka and Robyt 1998), are presently being used in the industry for their novel traits. Singh et al. (2009) conducted mutagenesis of *Leuconostoc dextranicum* NRRL B-1146 by UV irradiation and generated mutant strains with enhanced glucan production. Patel and Goyal (2010a) carried out UV mutagenesis on the natural isolate *P. pentosaceus* (Genbank Accession Number EU569832) and screened a novel mutant exhibiting higher dextransucrase activity. The wild type *P. pentosaceus* had an enzyme activity of 3.4 U/ml whereas the mutant showed 4.9 U/ml with 40% enhanced activity. The wild type *P. pentosaceus* had an enzyme activity of 3.4 U/ml whereas the mutant showed 4.9 U/ml with 40% enhanced activity. The wild type *P. pentosaceus* had specific activity of 0.58 U/mg whereas the mutant gave 1.0 U/mg showing 72% enhancement. The present study reports the comparative study of antibiotic resistance, carbohydrate fermentation, dextran synthesizing activity and stability of dextransucrase of wild type *P. pentosaceus* and its mutant.

## Material and methods

### Bacterial strains

The *P. pentosaceus* (PP) (Genbank Accession Number EU569832) isolate was screened from the soil sample collected from a sugarcane field of Assam (near Guwahati) (Patel and Goyal 2010a). Mutagenesis of *P. pentosaceus* was performed using UV irradiation. The colonies appeared on the UV-irradiated Petri plates were screened for their enzyme activity and specific activity. The mutant colony producing significantly higher dextransucrase than the wild type was selected for further study (Patel and Goyal 2010b). The stock cultures of wild type and mutant were maintained as MRS-S agar stab cultures at 4 °C and sub-cultured every 2 weeks (Goyal and Katiyar 1996)*.*

### Antibiotic sensitivity

The mutant of *P. pentosaceus* was tested for susceptibility to twenty seven antibiotics using agar disc diffusion test (Barry and Thornsberry 1980). The antibiotic tests were performed using commercially available antibiotic octodiscs containing Amoxyclav (Ac), Cephalexin (Cp), Ciprofloxacin (Cf), Clindamycin (Cd), Erythromycin (E), Ampicillin (A), Carbenicillin (Cb), Cephotaxime (Ce), Chloramphenicol (C), Co-Trimazine (Cm), Oxacillin (Ox), Amikacin (Ak), Amoxycillin (Am), Bacitracin (B), Cephalothin (Ch), Novobiocin (Nv), Oxytetracycline (O), Vancomycin (Va), Cephaloridine (Cr), Kanamycin (K), Lincomycin (L), Methicillin (M), Norfloxacin (Nx), Olaendomycin (Ol), PenicillinG (P), Tetracycline (T) and Gentamicin (G), from Hi-media Pvt. Ltd. India. MRS medium containing 2% glucose as carbohydrate source with 1.8% (*w/v*) agar and 0.8% (*w/v*) agar were used. The petri-plates were first prepared with MRS medium containing 1.8% (*w/v*) agar. The test strain was seeded in MRS-soft agar (0.8%, *w/v*) and overlaid in the Petri-plate having a bottom layer of above MRS agar (1.8%, *w/v*). The octodiscs were then gently placed over the surface of the seeded plate. The Petri plates were incubated in inverted position overnight in an incubator at 28 °C and were observed next day for zone of inhibition around the discs.

### Carbohydrate fermentation

The wild type and mutant of *P. pentosaceus* were tested to 13 different carbohydrates for their ability to ferment using the method of (Kandler and Weiss 1986). From the overnight grown MRS broth containing 2% glucose as carbohydrate source, 50 μl was inoculated in 5 ml liquid MRS medium lacking glucose but containing phenol red (0.04 g/L) as pH indicator and other test carbohydrates to give a final inoculum to medium ratio of 1% (*v/v*). The test media were incubated at static condition, for 48 h at 28 °C (Purama et al. 2008). The acid production was observed between 24 and 48 h. The acid production as a result of carbohydrate fermentation was indicated by a change in colour of phenol red to yellow.

### Culture conditions for dextransucrase production

For the development of inoculum, a loopful of culture from modified MRS agar stab was transferred to 5 ml of enzyme production medium as described by Tsuchiya et al. 1952. This enzyme production medium consists of (%, *w/v*) sucrose, 2; yeast extract, 2; K_2_HPO_4_, 2; MgSO_4_·7H_2_O, 0.02; MnSO_4_·4H_2_O, 0.001; FeSO_4_·7H_2_O, 0.001; CaCl_2_, 0.001; NaCl, 0.001 and the pH was adjusted to 6.9 (Tsuchiya et al. 1952). The wild type and mutant of *P. pentosaceus* cultures were incubated at 25 °C at 180 rpm for 12 h.

### Dextransucrase production from wild type and mutant of *P. pentosaceus*

One percent of the above 5 ml broth was again inoculated to 100 ml enzyme production medium contained in a 250 ml Erlenmeyer flask and incubated for 16 h at 25 °C under shaking at 180 rpm. One milliliter of broth sample was withdrawn and centrifuged at 10,000*g* for 10 min at 4 °C. The cell-free supernatant was analyzed for enzyme activity and protein concentration. All experiments were performed in duplicates for accuracy of results.

### Enzyme activity and protein concentration assay

The assay of dextransucrase activity was carried out in 1.0 ml of a reaction mixture in 20 mM sodium acetate buffer, pH 5.4, containing 146 mM (5%, *w/v*) sucrose and 20 μl cell-free supernatant as the enzyme source. The reaction mixture was incubated at 30 °C for 15 min. Aliquots (0.1 ml), from the reaction mixture were analyzed for reducing sugar concentration. The enzyme activity was determined by estimating the liberated reducing sugar by Nelson–Somogyi method (Nelson 1944; Somogyi 1945). The absorbance was measured at 500 nm using a UV-visible spectrophotometer (Cary 100 Bio, Varian, Inc., USA) against a blank using D-fructose as standard. One unit (U) of dextransucrase activity is defined as the amount of enzyme that liberates 1 μmol of reducing sugar per min at 30 °C in 20 mM sodium acetate buffer, pH 5.4. The protein concentration of the cell-free supernatant and other purified protein samples were estimated by the method of Lowry et al. (1951) using BSA as standard.

### Purification of dextransucrase with PEG fractionation

Dextransucrase used in the present study was purified by a single step fractionation method using polyethylene glycol (PEG) 400 (Purama and Goyal 2008). An ice cold PEG-400 solution was added to 100 ml cell-free extract at 4 °C, to get the final concentration 25% (*v/v*). The mixture was incubated at 4 °C for 12–16 h to allow dextransucrase to fractionate. The mixture was centrifuged at 13,000*g* at 4 °C for 30 min to separate the precipitated dextransucrase. The enzyme pellet was resuspended in 20 mM sodium acetate buffer (pH 5.4). The dextransucrase was subjected to dialysis using the same buffer and 5 kDa cutoff membrane to remove any trace of PEG-400. The purified dextransucrase obtained was analyzed for enzyme activity, protein concentration and purity by SDS-PAGE analysis.

### In situ detection of dextransucrase activity

For the detection of dextransucrase activity, periodic acid staining (PAS) of sucrose incubated gel on 7.5% non-denaturing SDS-PAGE (Holt et al. 2001) was done. Non-denaturing SDS-polyacrylamide gel electrophoresis was performed with a vertical slab mini gel unit (BioRad) using 1.5-mm thick gels (Laemmli 1970). After the run, the gel was treated thrice for 20 min with 20 mM sodium acetate buffer, pH 5.4 containing 0.005% (*w/v*) CaCl_2_ and 0.1% (*v/v*) Triton X-100 to remove the SDS at room temperature. The gel was then incubated in 10% sucrose solution in 20 mM sodium acetate buffer pH 5.4 for 6–8 h at 30 °C. Following incubation, the gel was washed twice with 70% (*v/v*) ethanol for 20 min and incubated in a solution containing 0.7% (*w/v*) periodic acid and 5% (*v/v*) acetic acid for 60 min at room temperature. The gel was again washed thrice with a solution containing 0.2% (*w/v*) sodium metabisulphite and 5% (*v/v*) acetic acid and was stained finally with Schiff’s reagent (0.5%, *w/v* Fuchsin basic, 1%, sodium bisulphite and 0.1 N HCl) until the discrete magenta bands within the gel matrix appeared, which confirmed dextransucrase activity. The other gel was stained with Coomassie Brilliant Blue for location of activity bands. Molecular mass marker proteins (myosin from rabbit muscle 205,000, phosphorylase b 97,400, bovine serum albumin 66,000, ovalbumin 43,000, carbonic anhydrase 29,000 Da) purchased from Genei, India, were used as standard for SDS-PAGE.

### Effect of stabilizers on stability of dextransucrase

Effect of stabilizers on stability of dextansucrase was studied by incubating the dextransucrase at different temperatures (30 and 4 °C). Aqueous solutions of dextran (100 kDa), PEG-8000, glycerol, Tween-80 were added to dextansucrase solution of wild type and mutant (0.24 mg/ml, 18 U/mg specific activity and 0.3 mg/ml, 18.2 U/mg) in sodium acetate buffer, pH 5.4 to obtain the final concentrations of dextran (100 kDa, 10 μg/ml), glycerol (0.5%, *v/v*), PEG 8000 (10 μg/ml) and Tween 80 (0.5%, *v/v*). The enzyme with or without any stabilizers was incubated at 30 °C for 24 h and 4 °C for 15 days (Purama et al. 2010). The aliquots (20 μl) were taken at indicated time intervals for the enzyme assay.

## Results and discussion

### Antibiotic susceptibility

A standardized filter-paper disc-agar diffusion assay allows a rapid determination of the efficacy of the drug by measuring the diameter of the zone of inhibition. The mutant of *P. pentosaceus* was tested for susceptibility to 27 antibiotics that represent the major antibiotic groups. Out of 27 antibiotics tested, the mutant displayed the significant differences in sensitivity and susceptibility to 9 antibiotics when compared with wild type as reported earlier (Patel and Goyal 2010b). In contrast to wild type, the mutant showed high or moderate sensitivity towards clindamycin, cephotaxime, amikacin, bacitracin, cephalothin, novobiocin, oxacillin and resistance against cephalexin, methicillin (Table 1).

**Table 1:** Antibiogram of wild type *P. pentosaceus* and its mutant of using antibiotic octodiscs on MRS agar

S. no.	Antibiotics	Conc.	PP wild type*	PP mutant
1.	Amoxyclav (Ac)	10 μg	M	M
2.	Cephalexin (Cp)	10 μg	M	R
3.	Ciprofloxacin (Cf)	10 μg	R	R
4.	Clindamycin (Cd)	2 μg	M	S
5.	Erythromycin (E)	15 μg	S	S
6.	Ampicillin (A)	10 μg	R	R
7.	Carbenicillin (Cb)	100 μg	S	S
8.	Cephotaxime (Ce)	30 μg	M	S
9.	Chloramphenicol (C)	30 μg	S	S
10.	Co-Trimazine (Cm)	25 μg	R	R
11.	Oxacillin (Ox)	5 μg	R	M
12.	Amikacin (Ak)	10 μg	R	S
13.	Amoxycillin (Am)	10 μg	S	S
14.	Bacitracin(B)	10 U	M	S
15.	Cephalothin (Ch)	30 μg	M	S
16.	Novobiocin (Nv)	30 μg	M	S
17.	Oxytetracycline (O)	30 μg	S	S
18.	Vancomycin (Va)	30 μg	R	R
19.	Cephaloridine (Cr)	30 μg	R	R
20.	Kanamycin (K)	30 μg	R	R
21.	Lincomycin (L)	2 μg	S	S
22.	Methicillin (M)	5 μg	S	R
23.	Norfloxacin (Nx)	10 μg	R	R
24.	Olaendomycin (Ol)	15 μg	S	S
25.	PenicillinG (P)	10 U	S	S
26.	Tetracycline (T)	30 μg	S	S
27.	Gentamicin (G)	10 μg	R	R

*R* resistant (0–0.1 cm), *M* moderate (0.2–0.8 cm), *S* sensitive (0.9–2.5 cm)

Values in centimeter are the distances of zone of inhibition of growth of microorganisms

* Patel and Goyal (2010a)

### Carbohydrate fermentation

The mutant was tested for its ability to ferment 13 carbohydrates and compared with wild type as reported earlier (Patel and Goyal 2010b). The critical nature of the fermentation and the activity of the indicator make it essential that all cultures should be observed within 48 h. Extended incubation may mask acid producing reactions by production of alkali because of enzymatic action on substrates other than the carbohydrate (Purama et al. 2008). The carbohydrate fermentation profile of both the wild type and mutant of *P. pentosaceus* was 62% similar. In carbohydrate fermentation profiling the mutant metabolized trehalose, galactose, maltose, lactose, and fructose with reduced efficiency as compared to wild type (Table 2).

**Table 2:** Carbohydrate fermentation profile of wild type *P. pentosaceus* and its mutant

S. No.	Carbohydrates	PP wild type*	PP mutant
1.	Xylose	−	−
2.	Trehalose	+++	++
3.	Mellibiose	+	+
4.	Galactose	++	+
5.	Mannitol	−	−
6.	Raffinose	−	−
7.	Cellobiose	+++	+++
8.	Rhamnose	+	+
9.	Maltose	+++	++
10.	Lactose	++	+
11.	Fructose	+++	++
12.	Glucose	+++	+++
13.	Sucrose	+++	+++

* Patel and Goyal (2010a)

(+++) strongly positive, (++) fairly positive, (+) weakly positive, (−) negative

### In situ detection of dextransucrase activity

**Fig. 1:**
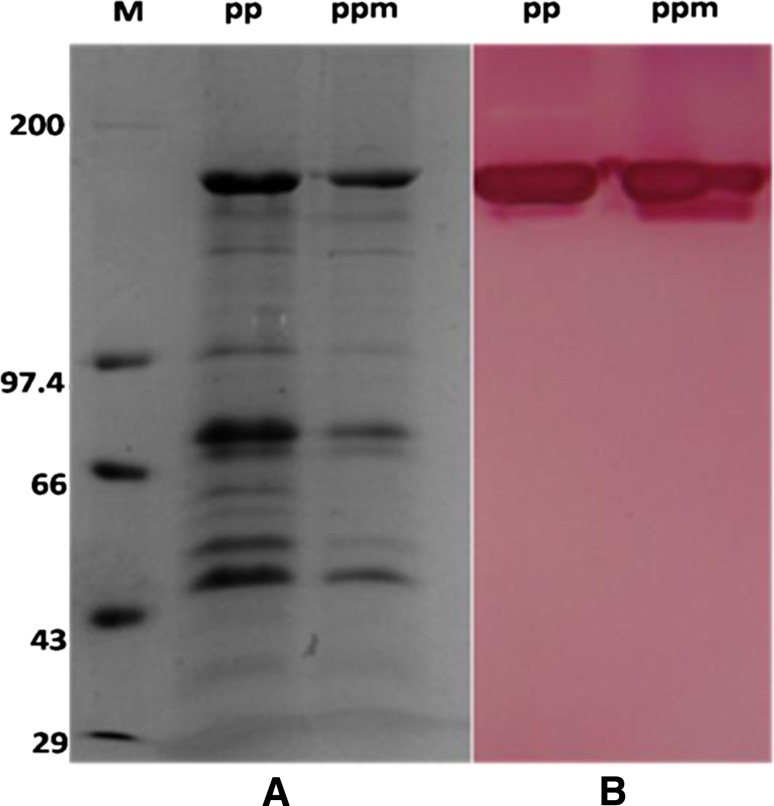
Identification of PEG purified dextransucrase from wild type and mutant of *P. pentosaceus*. **a** Lane M-Protein molecular weight marker (myosin from rabbit muscle 200 kDa, phosphorylase b 97.4 kDa, bovine serum albumin 66 kDa, ovalbumin 43 kDa, carbonic anhydrase 29 kDa); Lane pp-denaturing SDS-PAGE with Coomassie Brilliant Blue staining from wild type; Lane ppm-denaturing SDS-PAGE with Coomassie Brilliant Blue staining from mutant. **b** Lane pp- nondenaturing SDS-PAGE with Periodic Acid Schiff staining of the dextransucrase from wild type; Lane ppm-nondenaturing SDS-PAGE with Periodic Acid Schiff staining of the dextransucrase from mutant

Non-denaturing SDS-PAGE was used for in situ detection of enzyme activity to characterize dextransucrase production by wild type and mutant of *P. pentosaceus.* This study, however, was carried out to see if both the wild type and mutant of *P. pentosaceus* produce a similar or different dextran pattern that could be used to distinguish among the dextransucrase producing species (Purama et al. 2008). The results showed the presence of single protein band of approximately 180 kDa molecular size from both the wild type and mutant. The white bands were observed on the gels incubated in sucrose after 6–8 h. These white bands turn to magenta color after PAS staining, which confirmed the presence of dextran formed on polyacrylamide gels (Fig. 1b). The PAS staining of the sucrose incubated gels showed that the activity bands corresponded to the native and active form of the purified enzyme of approximately 180 kDa molecular size appearing on the denaturing gels stained with Coomassie brilliant blue (Fig. 1a).

### Effect of stabilizers on stability of dextransucrase

**Fig. 2:**
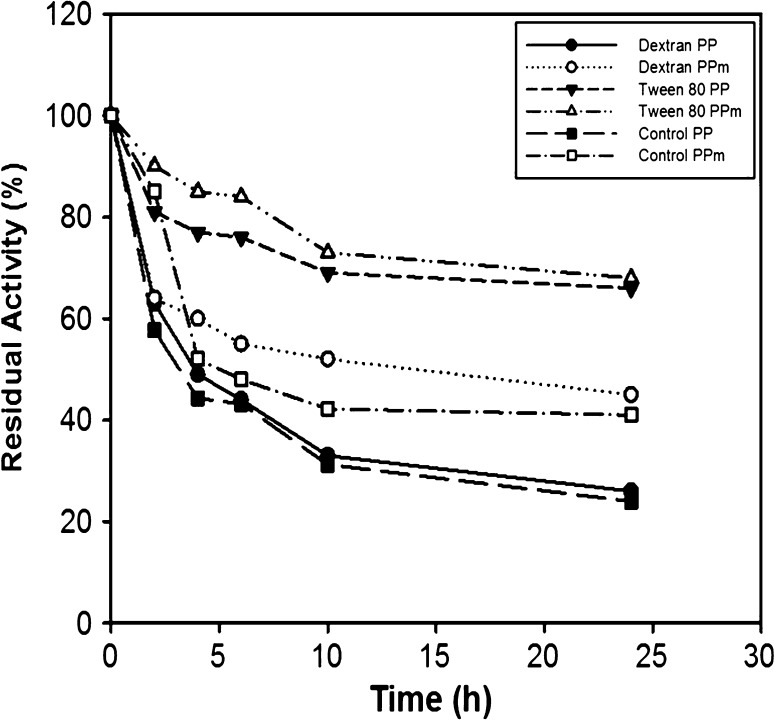
Effect of various stabilizers on dextransucrase activity from wild type and mutant of *P. pentosaceus* at 30 °C

**Fig. 3:**
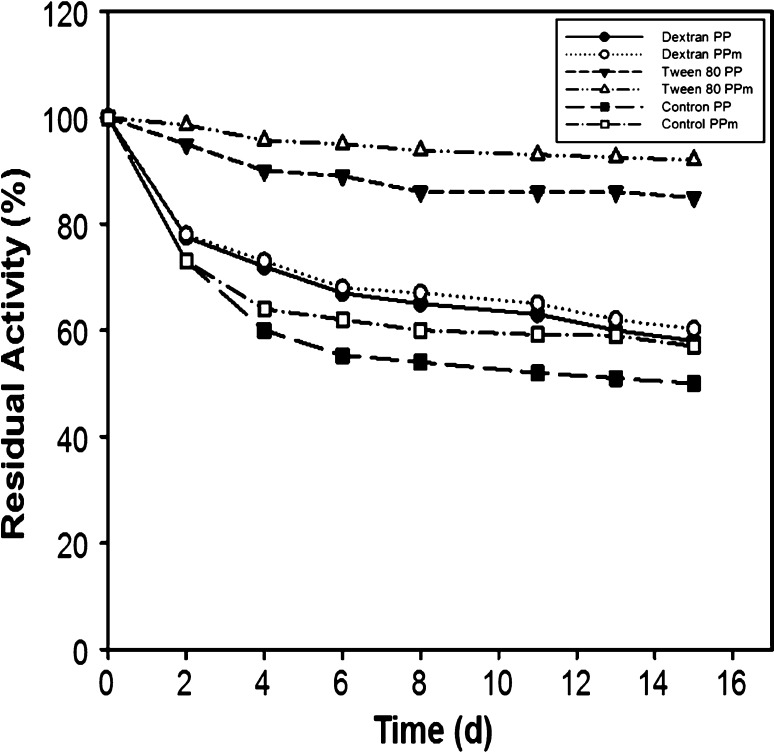
Effect of various stabilizers on dextransucrase activity from wild type and mutant of *P. pentosaceus* at 4 °C

The effects of various stabilizers on the stability of dextransucrase from the wild type and mutant of *P. pentosaceus* were studied at 30 and 4 °C. Both enzymes were more stable at 4 °C (Fig. 3). The residual activity of dextransucrase with Tween 80, PEG 8000, dextran (100 kDa), glycerol and without any stabilizer at 30 °C at 24 h was 66, 24, 26, 19 and 26% for wild type and 68, 28, 45, 38 and 41% for mutant of *P. pentosaceus,* respectively (Fig. 2). The residual activity of dextransucrase with Tween 80, PEG 8000, dextran (100 kDa), glycerol and without any stabilizer at 4 °C on 15th day was 85, 47, 58, 40 and 50% and 92, 48, 60, 53 and 57% for wild type and mutant of *P. pentosaceus* respectively (Fig. 3). The other stabilizers dextran (100 kDa), glycerol and PEG-8000 did not show any significant effect at both the temperatures on the enzyme. The data for glycerol and PEG-8000 are not shown in Figs. 2 and 3. It has been reported earlier that dextransucrase is stable at lower temperatures (10–30 °C) and loses rapidly the enzyme activity at temperatures higher than 30 °C (Purama et al. 2010). Our results are similar to those reported earlier for *L. mesenteroides* NRRL B-640 (Purama et al. 2010). The results clearly indicate that the mutant enzyme is more stable than the wild type enzyme and Tween 80 was the best stabilizer for dextransucrase of both the wild type and mutant of *P. pentosaceus.*

### Half-life of stabilizer treated dextransucrase

The residual activity of dextransucrase was measured at various temperatures with respect to time with and without stabilizers. The enzyme deactivation followed first-order rate kinetics. The half-life (*t*_1/2_) of dextransucrase and stabilizers treated dextransucrase was calculated by assuming that the decay followed first-order kinetics (Purama et al. 2010). Amongst all the stabilizers Tween 80 displayed maximum stabilization of dextransucrase with *t*_1/2_ of 28.5 and 33.5 h for wild type and mutant, respectively, at 30 °C and *t*_1/2_ of 52.1 and 106.6 days for wild type and mutant respectively, at 4 °C. The addition of Tween 80 to dextransucrase, incubated at both the temperatures (30 and 4 °C) resulted in higher *t*_1/2_ than that of with no Tween 80 (Table 3). The *t*_1/2_ of mutant enzyme was 49% higher and 24% higher than that of wild type at 30 and 4 °C, respectively. The *t*_1/2_ of dextransucrase from mutant with Tween was 17.5% higher and 104.6% higher than that of wild type at 30 and 4 °C, respectively. Taken together all these results, it can be summarized that Tween 80 provided the maximum stabilization at 30 and 4 °C and the mutant showed better stabilization than that of the wild type at both the temperatures.

**Table 3:** Half-life of dextransucrase from wild type *P. pentosaceus* and mutant of at 30 and 4 °C

Stabilizers	30 °C		4 °C	
	PP wild type *t*_1/2_ (h)	PP mutant *t*_1/2_ (h)	PP wild type *t*_1/2_ (days)	PP mutant *t*_1/2_ (days)
Control	8.50	12.70	11.76	14.53
Glycerol	7.50	12.25	10.40	14.00
PEG 8000	8.36	10.22	11.03	11.21
Tween 80	28.52	33.50	52.10	106.61
Dextran	8.51	15.23	16.04	17.11

## Conclusion

The comparison of antibiotic resistance, carbohydrate utilization pattern, dextransucrase activity and dextransucrase stabilization of wild type and mutant of *P. pentosaceus* was reported. The results of antibiotic resistance, carbohydrate utilization pattern, dextransucrase activity and dextransucrase stabilization will enhance understanding of these industrially significant species and will aid in distinguishing between physiologically similar species. The data will be useful for industrial applications where the strains are required with higher enzyme stability. Both dextransucrase of wild type and mutant were more stable at 4 °C than at 30 °C. Amongst various stabilizers Tween 80 provided the maximum stabilization to dextransucrase against activity loss at 30 and 4 °C. The addition of Tween 80 to dextransucrase at 30 and 4 °C resulted in higher *t*_1/2_ than that of without Tween 80. The residual activity and *t*_1/2_ were higher for mutant enzyme than that of wild type. The results suggested that dextransucrase from the mutant showed better stabilization than that of the wild type and therefore have greater importance.
